# Heat-Killed Tobacco Mosaic Virus Mitigates Plant Abiotic Stress Symptoms

**DOI:** 10.3390/microorganisms11010087

**Published:** 2022-12-29

**Authors:** Sruthy Maria Augustine, Stavros Tzigos, Rod Snowdon

**Affiliations:** Department of Plant Breeding, IFZ Research Center for Biosystems, Land Use and Nutrition, Justus Liebig University, Heinrich Buff Ring 26, 35392 Giessen, Germany

**Keywords:** drought stress tolerance, heat killed virus, tobacco mosaic virus

## Abstract

Since the discovery of the tobacco mosaic virus in the 1890s, awareness has grown in regard to how viruses affect the environment. Viral infections are now known to cause various effects besides pathogenicity, with some viruses in fact having a beneficial impact on plants. Although research has focused on disease-causing viruses that can infect plants, many wild plants are also infected with non-pathogenic viral agents. Traditionally, abiotic, and biotic stresses have been studied as isolated stimuli that trigger signaling pathways within the plant. However, both biotic and abiotic stress can trigger complex molecular interactions within plants, which in turn drive interconnected response pathways. Here, we demonstrate that heat-killed tobacco mosaic virus (TMV) can increase abiotic stress tolerance in plants, an effect that could potentially be implemented in challenging growth environments. To our knowledge, this is the first report of plant abiotic stress tolerance following treatment with heat-killed viral particles.

## 1. Introduction

Global warming, one of the biggest threats to sustainable agriculture, can increase the severity of yield losses and other adverse effects caused by biotic and abiotic stresses on crop production. Global warming is mainly caused by the release of greenhouse gases into the atmosphere, which results in a warmer climate. This can result in a serious threat to the environment that affects various aspects of society. It can cause plants to become vulnerable to various stresses and strains that eventually affect crop production. The two most common abiotic stresses, heat and drought, can stimulate plant pathogens such as fungi and viruses. These interactions can further affect the plant’s ability to adapt to environmental fluctuations [1]. In nature, plants are exposed to various combinations of abiotic and biotic stresses. Different kinds of stress result in similar kinds of stress response signals, which may share multiple nodes that can help the plant simultaneously adapt to complex environmental conditions [2,3,4]. Plant species have evolved effective strategies to survive harsh environmental conditions, utilizing coordinated signaling and response systems to respond efficiently to varying levels of stress. However, many crops are poorly adapted to the increasing frequency of extreme stress scenarios resulting from climate change. Plant viruses have historically been viewed as strictly pathogenic agents, causing plant injury and economic losses for producers [5]; however, viral infections are much more widespread in managed and unmanaged systems than previously thought and can cause a variety of effects other than pathogenicity [6]. 

Theoretical and scientific studies have shown that viruses are not solely destructive agents but also important components of the ecosystem [1,7]. Abiotic stresses can also affect the life cycle of viruses and their interactions with host defenses [7]. Different types of viruses, which can be broadly categorized into different genera, have been known to establish beneficial relationships with their hosts under abiotic stress [7]. Viral infection has been found to trigger resistance responses, which can help protect plants from abiotic stress [8,9]. Many RNA viruses, including the cucumber mosaic virus, the tobacco mosaic virus (TMV), and the bromo mosaic virus, can affect various crops besides the direct hosts they are named after, for example, rice and beet. [8] found that these viruses could delay the appearance of drought-related symptoms in their hosts. Interactions between viruses and hosts can lead to a spectrum of mutualism and pathogenesis, that can switch throughout the lifecycle. The reduction in transpiration rate that can be observed under viral infection is often linked to the development of drought tolerance [8,10,11]. Infection by the tomato yellow leaf curl virus, a DNA virus, has been shown to confer drought resistance and thermotolerance on tomato plants [1,12,13,14]. Metabolite profiling has revealed that viral infections can help plants tolerate abiotic stresses by increasing their levels of antioxidants and osmoprotectants [8]. For example, using microarray and metabolite profiling, [15] showed a virus-specific shift in Arabidopsis signaling networks under drought and heat stress in combination with virus infection.

The development of drought tolerance in *Nicotiana benthamiana* and *Arabidopsis thaliana* infected with potato virus X (PVX) and plum poxvirus (PPV) was associated with the presence of increased levels of salicylic acid (SA) but not abscisic acid (ABA) in the plants. Although a detailed description of the effects of these viruses on plant survival was not provided, the presence of these nutrients in the plants was detected [10]. In the present study, we evaluated the effect of TMV-infected and heat-killed TMV introduced on tobacco plants under drought and heat stress. We analyzed the cell membrane stability, relative water content, chlorophyll content, and expression of stress-responsive genes. To the best of our knowledge, this is the first evaluation of drought stress responses in plants treated with HKTMV.

## 2. Materials and Methods

### 2.1. Plants and Treatment Conditions

Dr. Ulrich Commandeur, RWTH Aachen University, kindly provided the U1 strain TMV inoculum. 

Wild-type tobacco plants (*Nicotiana tabacum* cv. Petit Havana SR1) (3–4 weeks old, when the plants had reached the 2–3 leaf stage) [16] were inoculated following the method described in [17,18]. The TMV inoculated and uninoculated plants were cultivated in two different growth chambers (CLF plant climatics; E-36HO) (the photoperiod was 16 h of light, 8 h of darkness, 5000–10,000 lux, and 26 °C/20 °C for light/dark temperature, respectively). After inoculation, the TMV infection symptoms appeared in 7–14 days, and drought stress started on the 15th day by withholding water for a period of 10 days with a 35 °C/30 °C light/dark temperature regime. The photoperiod was 16 h of light and 8 h of darkness.

The heat-killed TMV (HKTMV) was prepared using a 5 µM TMV virus inoculum in PBS buffer and heat-killed at 1000 ℃ for 90 s, followed by 650 ℃ for 10 min. The heat killed the TMV virus, which was inoculated into the plants using the method described in [17,18]. After inoculation, the plants were observed for any TMV infection symptoms, and drought stress was started on the 15th day by withholding water for a period of 10 days with a 35 °C/30 °C light/dark temperature regime. The photoperiod was 16 h of light and 8 h of darkness. 

### 2.2. Cell Membrane Thermostability Analysis

The cell membrane stability (CMS) test estimated the percentage of cell membrane injury as previously described [19] and is an indicator of drought tolerance [20]. This parameter was studied in TMV-inoculated plants as well as uninoculated control plants. The third fully opened leaf was collected from TMV inoculated and uninoculated control plants on days 0 and 10 after the application of drought and heat stress. Leaf discs (0.5 cm in diameter) weighing 200 mg were washed 3 times for 2 min with 20 mL of distilled water. The leaf discs were then immersed in 20 mL distilled water in 2.5 cm × 15 cm tubes covered with aluminum foil and incubated at 60 °C in a thermostatically controlled water bath for 20 min before cooling to 10 °C for 12 h to allow the diffusion of electrolytes. An initial conductance reading was taken at 30 °C using a conductivity meter, then the tubes were heated to 100 °C for 20 min, and a second conductance reading was taken after cooling to 30 °C. Membrane injury % = 1 − ((1 − T_1_/T_2_)/(1 − C_1_/C_2_)) × 100, where T and C refer to the values for treatment and control samples, respectively, and the subscripts 1 and 2 denote the initial and final conductance readings, respectively.

### 2.3. Plant Water Status

The relative water content (RWC) of excised third leaves from TMV-inoculated and uninoculated control plants at the 4–6 leaf stage was determined on days 0 and 10 after the induction of drought stress. The RWC was calculated based on the fresh weight (FW), turgid weight (TW), and dry weight (DW) of 200 mg leaf samples. The FW was determined based on a mass balance immediately after sample collection. The TW was determined after soaking the leaf discs in deionized water for 4 h at room temperature in a closed Petri dish and then blotting off any surface drops. The DW was determined after oven drying at 90 °C for 72 h. The RWC was determined as previously described [21] using the following equation:

RWC = ((FW − DW) / (TW − DW)) × 100(1)

### 2.4. Leaf Chlorophyll Content

The chlorophyll content was estimated with a Chlorophyll Content Meter CCM-200 from Opti-Sciences. The CCM-200 is a hand-held instrument designed for the rapid, nondestructive determination of the chlorophyll content of intact leaf samples. The CCI was determined following the method described in [22]. The CCI was determined on the third fully opened leaf from TMV-inoculated and uninoculated control plants on days 0 and 10 after the application of drought and heat stress. 

### 2.5. RT-PCR

Total RNA was isolated from leaf samples using RNAsolv (Omega Bio-Tek, Norcross, GA, USA), followed by treatment with DNase (Thermo Fisher Scientific, Waltham, MA, USA). First-strand cDNAs were synthesized from total RNA using the Revert Aid first-strand cDNA synthesis kit and oligo (dT) primers (Thermo Fisher Scientific). The primers F-5′-CGACATCAGCCGATGCAGC-3′ and R-5′-ACCGTTTTCGAACCGAGACT-3′ were used to amplify the TMV virus, which results in an 880 bp PCR product. The following conditions were used: a temperature of 95 °C for 2 min, followed by 30 cycles at 95 °C for 30 s, 58 °C for 30 s, and 72 °C for 30 s, then a final extension step at 72 °C for 7 min before cooling to 4 °C.

### 2.6. Gene Expression Analysis Using the Comparative CT Method

Total RNA was isolated from leaf samples using RNAsolv (Omega Bio-Tek) [23] followed by treatment with DNase (Thermo Fisher Scientific). First-strand cDNAs were synthesized from total RNA using the Revert Aid first-strand cDNA synthesis kit and oligo (dT) primers (Thermo Fisher Scientific) with ADP-ribosylation factor as an internal control. Gene-specific primers designed using Primer Express v3 (Applied Biosystems, Waltham, MA, USA) were then used for 40 cycles of specific amplification. Each reaction comprised 12 µL of SYBR green master mix (Thermo Fisher Scientific) and 10 pmol of each gene-specific primer and was carried out on a Step One real-time PCR system (Applied Biosystems). The CT values for both the target and internal control genes were used to quantify the transcripts by comparative CT normalization. All reactions were performed in triplicate, and the expression of the target gene was calculated using the formula 2^–∆∆Ct^ ((Ct gene of interest—Ct internal control) sample—(Ct gene of interest—Ct internal control) control) [24]. The ∆∆Ct values reflect the relative expression of the target gene following exposure to osmotic stress.

### 2.7. Statistical Analysis

For statistical analysis of the data, three biological and technical replications were used, and the experiment was repeated three times. The mean value and standard deviation were evaluated using the XLSTAT 2013.5 program to analyze all the data and compare the TMV-inoculated, HKTMV-inoculated, and uninoculated control plants under normal and stress conditions. Unpaired Student’s *t*-test was used to analyze the statistical significance among treatment and control groups. The Student’s *t*-test was performed using GraphPad Prism. 


**Gene**

**Primer Sequences**
ADP-ribosylation factorF-5′-TTCGGCAAGCTTTTCAGTCG-3′ R-5′-TCCCTGGGTGTTTTGGAAGT-3′Hsp70F-5′-CGGTAACCCAAGAGCCCTTA-3′ R-5′-TCAACGGGCTCCATACACTT-3′DREB2F-5′-TGCAACATACAGGGGAGTGA-3′ R-5-TCTGCAGTGGGGTAAGTTCC-3′WRKY1F-5′-CGCAAGGCCTGAGAAAACTT-3′ R-5′-CCCGTCATGTGATCTCTCCA-3′ERF1BF-5′-GCCATGGGGTAAATATGCAG-3′ R-5′-AGCAGCAGGAGACAATCCAT-3′ADFF-5′-TTCTGGCATGGGTGTAGCTG-3′ R-5′-GCTGCCAGTTTTCTCAACAA-3′

## 3. Results and Discussion

**Figure 1:**
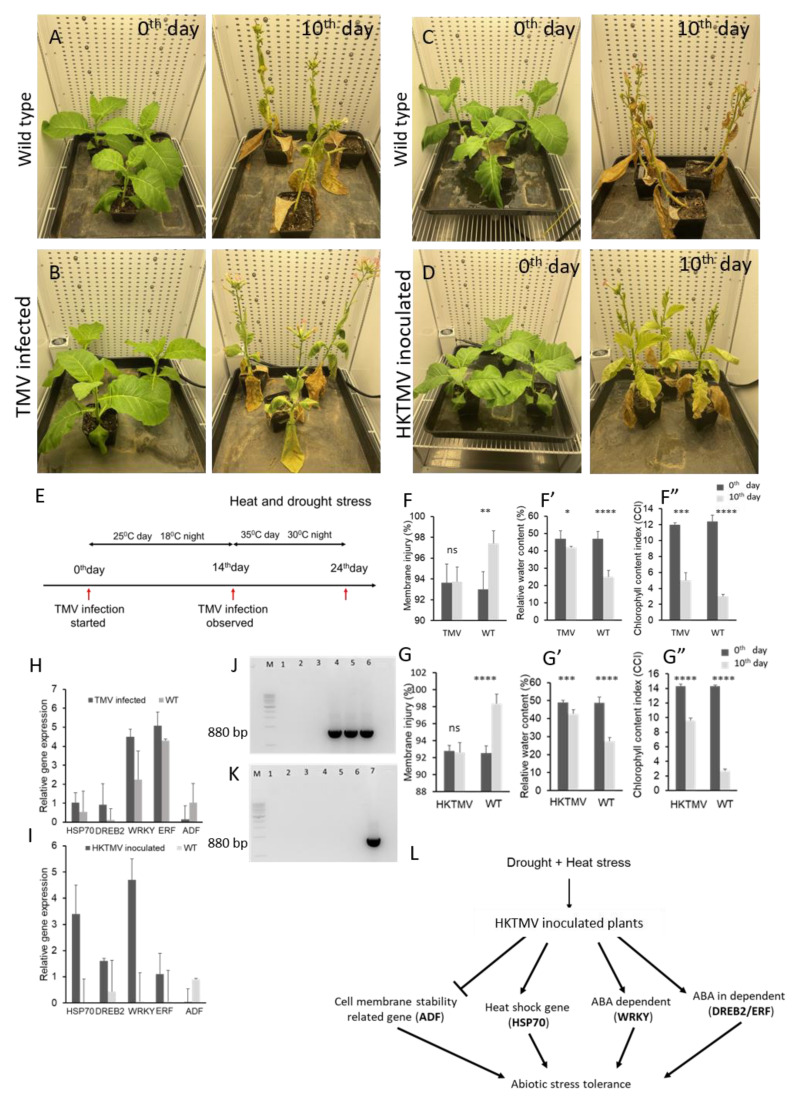
(**A**) Uninoculated control plants on days 0 and 10 of stress. (**B**) TMV-inoculated plants on days 0 and 10 of stress. (**C**) Uninoculated control plants on days 0 and 10 of stress. (**D**) HKTMV inoculated plants on day 0 and 10 of stress. (**E**) Schematic representation of the treatment procedure. (**F**) Membrane thermostability of TMV-inoculated and control plants on days 0 and 10 of drought and heat stress. Data labeled with ** *p* < 0.05 show a significant difference by the Student’s *t*-test. (**F’**) Relative water content of TMV-inoculated and control plants on days 0 and 10 of drought and heat stress. Data labeled with * *p* < 0.05 and **** *p* < 0.0001 show a significant difference by the Student’s *t*-test. (**F”**) Chlorophyll content index of TMV-inoculated and control plants on days 0 and 10 of drought and heat stress. Data labeled with *** *p* < 0.001 and **** *p* < 0.0001 show a significant difference by the Student’s *t*-test. (**G**) Membrane thermostability of HKTMV-inoculated and control plants on days 0 and 10 of drought and heat stress. Data labeled with **** *p* < 0.0001 show a significant difference by the Student’s *t*-test. (**G’**) Relative water content of HKTMV-inoculated and control plants on days 0 and 10 of drought and heat stress. Data labeled with *** *p*<0.001 and **** *p* < 0.0001 show a significant difference by the Student’s *t*-test. (**G”**) Chlorophyll content index of HKTMV-inoculated and control plants on days 0 and 10 of drought and heat stress. Data labeled with **** *p* < 0.0001 show a significant difference by the Student’s *t*-test. (**H**) Relative expression of TMV-inoculated and uninoculated control plants on days 0 and 10 of drought and heat stress using a comparative CT method. (**I**) Relative expression of HKTMV-inoculated and uninoculated control plants on days 0 and 10 of drought and heat stress using the comparative CT method. (**J**) Analysis of the TMV infection using RT-PCR lane M—1 Kb DNA ladder, lanes 1–3—uninoculated control plants, and lanes 4–6—TMV-inoculated plants. (**K**) Analysis of the absence of live TMV using RT-PCR lane M—1 Kb DNA ladder, lanes 1–3—uninoculated control plants, lanes 4–6—HKTM- inoculated plants, and lane 7—positive control. (**L**) Schematic representation of the mechanism of heat-killed virus-induced abiotic stress tolerance in plants.

We inoculated tobacco plants (*Nicotiana tabacum* cv. Petit Havana SR1) with live tobacco mosaic virus (TMV) from the 4 to 6 leaf stage. Viral infection symptoms developed in 10–14 days, and the presence of the virus was confirmed using reverse transcriptase polymerase chain reaction (RT-PCR) (Figure 1J). After confirming the presence of the virus, drought and heat stress were applied for a period of 10 days to virus-infected and uninfected control plants in two different climate-controlled growth chambers. We observed that virus-infected plants were more tolerant to drought and heat stress than uninfected control plants (Figure 1A,B). A schematic representation of the treatment procedure is given in Figure 1E. The stress tolerance was analyzed by measuring physiological parameters, including cell membrane thermostability, relative water content (RWC), and chlorophyll content index, along with expression levels of known stress-response genes. Interestingly, virus-infected plants exhibited higher membrane stability and relative water content under drought and heat stress than uninfected control plants (Figure 1F,F’). Membrane stability, a widely accepted measure of drought tolerance, was measured by using a conductivity meter to assess membrane injury. The results suggested that virus-infected plants reacted to a decline in soil moisture with better maintenance of cell membrane integrity compared to non-infected control plants, suggesting that the virus infection improves the plant’s ability to adjust to an increase in stress. The water content in the virus-infected plants was also much higher than that in the uninfected control plants under drought and heat stress (Figure 1F’). A sharp decline in the chlorophyll content index (CCI) was observed in both virus-infected and uninoculated control plants under stress (Figure 1F”). The virus-infected plants, although they showed higher tolerance to stress, displayed a lower chlorophyll content in the presence of TMV. Under normal irrigation, both the control and treatment groups showed similar membrane injury levels, water content, and chlorophyll content, respectively (Figure 1F,F’,F”). Under drought and heat stress, however, the TMV-inoculated plants showed significantly higher membrane stability and water content than the uninoculated control plants (Figure 1F,F’). Furthermore, we analyzed expression levels of well-known stress-responsive genes known to be required for plant survival in compromised environments. The abiotic stress-responsive genes dehydration-responsive element binding protein (DREB2), heat shock protein (Hsp70), WRKY transcription factors (WRKY1,) and ethylene response factor (ERF1B) were highly expressed in TMV-infected plants compared to the uninoculated control (Figure 1H) under both drought and heat stress. We previously demonstrated that downregulation of the gene actin-depolymerizing factor (ADF) increases drought stress tolerance in sugarcane and tobacco by enhancing cell membrane stability [25,26]. Here, we found that ADF was downregulated in TMV-infected plants compared to the uninoculated control (Figure 1H). The TMV-infected plants showed a 1-fold increase in DREB2 and Hsp70, a 5-fold increase in ERF1B, and a 4.5-fold increase in WRKY1 expression, respectively, compared to uninfected control plants. After withholding water, drought symptoms appeared in control plants in 2–3 days, whereas virus-infected plants delayed the drought symptoms for 5–6 days. Our results suggest that the TMV-infected plants had higher adaptability under severe stress than the uninfected control plants (Figure 1B). However, TMV-infection symptoms in the plants, for example yellowing veins and malformed leaves, had a negative effect on growth and biomass, which can hinder the use of TMV in crop protection programs.

Studies in animals also showed that heat-killed viral particles can induce an immune response [27], and a heat-killed entophytic bacterium was found to induce pathogen defense responses in Arabidopsis [28]. Here we inoculated tobacco plants with heat-killed tobacco mosaic virus (HKTMV) from the 4 to 6 leaf stage using mechanical aberration, and viral infection symptoms were monitored for 14 days. On the second day, few yellow spots were observed. While plants looked healthy, approximately 5 days later with no signs of infection and elevated chlorophyll content (Figure 1D,G”). Drought and heat stress were induced in two different environment-controlled climate chambers for HKTMV-inoculated and uninoculated control plants by withholding water for a period of 10 days, and the temperature was maintained at 35 °C during the daytime and 30 °C during the night. Plants were analyzed on day 0 prior to stress application and on day 10 after drought and heat stress application. Our data showed that treatment with heat-killed viral particles protected plants from severe drought and heat stress (Figure 1C,D). To our knowledge, this is the first report of plants acquiring enhanced drought and heat stress tolerance after treatment with a heat-killed virus. The absence of the live virus was confirmed using RT-PCR (Figure 1K). Cell membrane stability was tested on day 0 before the stress treatment and on day 10 of drought and heat stress. The HKTMV-inoculated plants showed a stable cell membrane under drought and heat stress compared to the uninoculated control plants. As the soil moisture declined, the cell membrane stability was not changed in HKTMV-inoculated plants, but there was a significant reduction in membrane stability in the uninoculated plants (Figure 1G). In addition, the water content is significantly reduced in uninoculated control plants compared to HKTMV-inoculated plants under drought and heat stress (Figure 1G’). Chlorophyll content was only mildly affected in the HKTMV-inoculated plants compared to the uninoculated and TMV-infected plants (Figure 1G”). The data suggest that heat-killed viral particles can alter the downstream mechanisms of drought and heat stress and impart stress tolerance. The stress-response genes DREB2, Hsp70, WRKY1, and ERF1B were highly expressed in HKTMV-inoculated plants compared to uninoculated plants (Figure 1I), while ADF was downregulated in HKTMV-inoculated plants compared to uninoculated control plants (Figure 1I). HKTMV-infected plants showed a 1.6-fold increase in DREB2, a 3.4-fold increase in Hsp70, a 1.1-fold increase in ERF1B, and a 4.7-fold increase in WRKY1 expression compared to uninoculated control plants. These results suggest that an application of HKTMV might potentially protect plants by modulating the expression of important genes involved in plant survival under stress. In comparison to TMV infection, HKTMV appears to mitigate the severe effects of drought and heat stress without affecting plant growth and morphology; plants were able to maintain higher chlorophyll contents, showed superior drought and heat stress tolerance, and showed no symptoms of viral infection (Figure 1L). Further in-depth analysis is needed to reveal the molecular players and mechanism behind HKTMV-induced abiotic stress tolerance. Meanwhile, in the face of global climate change, HKTMV treatments might represent a potential option to mitigate the impact of heat and drought stress on crop production. 

Plants have developed a defense system that allows them to respond efficiently to varying environmental conditions. Understanding these natural protection strategies is a major challenge. Defense priming can be utilized to improve the plant’s defense mechanisms against abiotic stresses through the introduction of biological and chemical priming agents [29]. In this study, we used heat-killed viruses as priming agents. Viruses normally parasitize host resources for their own reproduction and are therefore generally considered harmful to plants. Understanding the drought response mechanism triggered by the virus infection will provide valuable insight into the role of these organisms in the ecology and evolution of the host. However, we observed that the presence of heat-killed viruses could improve plant survival under harsh environmental conditions without causing any viral infection symptoms. A delay in the onset of drought-stress symptoms can have a very significant impact on plant survival and performance under stress. Heat-killed viruses are therefore potentially useful for various agricultural applications because drought is one of the most critical factors affecting the production of crops worldwide. The preliminary results presented in this study indicate that heat-killed viruses might be suitable for use as a protecting agent for plants from the severe effects of abiotic stress.
